# Evaluation of phenol treatment for pilonidal sinus in adolescents

**DOI:** 10.3389/fped.2025.1595749

**Published:** 2025-05-19

**Authors:** Ayten Başak Kılıç, Merve Nur Muti Acar, Sinan Kılıç, Gülşen Ekingen

**Affiliations:** ^1^ Department of Pediatric Surgery, Elite Medical Center, Doha, Qatar; ^2^ Department of Pediatric Surgery, Private Ilgi Medical Center, Gebze, Türkiye; ^3^ Faculty of Medicine, Department of Pediatric Surgery, Okan University, Tuzla, Turkiye; ^4^ Faculty of Medicine, Department of Pediatric Surgery, Kocaeli University, Kocaeli, Turkiye

**Keywords:** pilonidal sinus disease, phenol treatment, adolescents, minimally invasive surgery, recurrence

## Abstract

**Objective:**

Pilonidal sinus disease (PSD) is chronic condition predominantly affecting adolescents, often associated with risk factors such as obesity, poor hygiene, and prolonged sitting. Minimally invasive treatments like phenol procedure have gained attention as alternatives to traditional surgical methods due to their simplicity and potential for shorter recovery. This study aims to evaluate the efficacy and safety of phenol procedure in adolescents with pilonidal sinus, focusing on success rates, recurrence, and complications.

**Material and method:**

Total number of 28 adolescent patients with non-complicated PSD were included in this retrospective study.

**Results:**

This study included 28 adolescent patients (mean age: 15.04 ± 1.40 years), of which 60.7% were female. A total of 46.4% of the patients presented with a single sinus opening, while 53.6% had multiple sinus openings. Phenol procedure was administered with a mean of 3.32 ± 1.49 courses, resulting in complete healing in 75.0% of patients and an overall success rate of 82.1% after pre- or post-surgical procedure. Complications were observed in 14.3% of cases, while recurrence occurred in 21.4%. Persistent discharge was reported in 7.1% of patients, necessitating surgical excision.

**Conclusions:**

Our findings support that phenol treatment is a safe and effective minimally invasive approach for managing pilonidal sinus, demonstrating success rates comparable to conventional surgical methods. Furthermore, phenol procedure offers notable advantages, including preservation of tissue integrity, ease of implementation, and reduced recovery times, rendering it particularly advantageous for adolescent patients.

## 1 Introduction

Pilonidal sinus disease (PSD) is a chronic inflammatory condition of the intergluteal region, most commonly affecting adolescents and young adults. Hair penetration into the skin triggers a granulomatous reaction, which can lead to abscess formation in some cases (1). It is considered an acquired condition, likely developing due to hair follicle obstruction. PSD is associated with obesity, hirsutism, a sedentary lifestyle, and local irritation. Among adolescents, prolonged sitting on hard surfaces and inadequate hygiene are key risk factors (2). The disease has been reported at rates of 26 per 100,000 in the United States and 48 per 100,000 in Germany (3).

Phenol procedure, a minimally invasive treatment for pilonidal sinus, has gained traction due to its simplicity, cost-effectiveness, and reduced complication rates compared to surgical interventions. This procedure involves the chemical destruction of sinus tracts, promoting healing without extensive excision or the need for prolonged wound care. Literature indicates that phenol procedure has demonstrated comparable success rates to surgical excision, with advantages such as shorter recovery times and lower rates of recurrence, particularly in adolescent populations (4). Furthermore, studies have suggested that phenol procedure is less invasive, preserving the option for future surgical intervention if required (5).

This study hypothesizes that phenol procedure is a safe and effective treatment for PSD in adolescents, offering success rates comparable to surgical interventions while reducing recurrence and postoperative complications. Therefore, this study aims to evaluate the efficacy and safety of phenol procedure in adolescents with PSD, focusing on success rates, recurrence, and associated complications.

## 2 Materials and Methods

### 2.1 Study design and patient selection

This retrospective study was approved by the Kocaeli University Faculty of Medicine Ethics Committee (Project No: 2025/74, Approval No: E-80418770-020-751658).

A total of 28 adolescent patients (aged 10–18 years) diagnosed with non-complicated PSD were included. Exclusion criteria comprised prior surgical interventions, recurrent cases, acute abscess formation, autoimmune diseases, and hidradenitis suppurativa.

Demographic data, including age, gender, BMI, symptom duration, and the number of sinus openings, were recorded at the initial assessment. Written informed consent was obtained from all participants before their inclusion in the study.

### 2.2 Preoperative preparation

Before the procedure, patients were advised to use depilatory creams or other forms of hair removal, such as laser hair removal, depending on personal preference.

Patients were positioned in the prone position. The natal cleft area was shaved, and the skin was disinfected with povidone-iodine. Local anesthesia was administered using 2–6 ml of bupivacaine or lidocaine. Preoperative bowel preparation and antibiotic prophylaxis were not required for non-infected cases. However, patients with infected pilonidal disease received appropriate antibiotic therapy before the procedure.

### 2.3 Procedure

The pilonidal sinus tracts were identified using a metal surgical clamp. Both primary and secondary openings were marked. The openings were then enlarged with the same clamp, and debris such as hair and necrotic tissue was removed through curettage.

**Figure 1:**
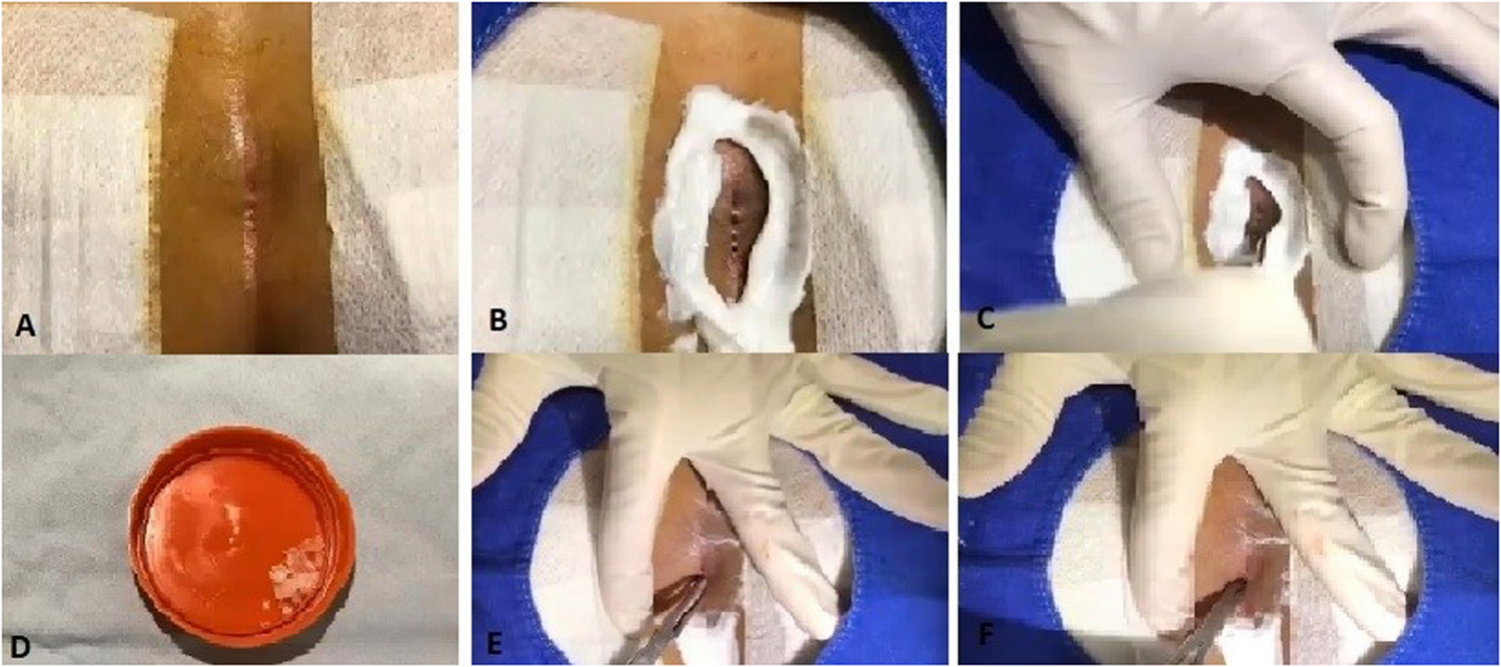
Sequential steps of the crystallized phenol procedure for pilonidal sinus disease. **(A)** Preoperative visualization of the sinus openings. **(B)** Application of a barrier cream to the surrounding healthy skin. **(C)** Mechanical dilation of the sinus openings using a surgical clamp. **(D)** Appearance of crystallized phenol in its solid form. **(E)** Insertion of crystallized phenol into the pilonidal sinus cavity. **(F)** Waiting period to allow phenol action, followed by application of a sterile dressing.

**Figure 2:**
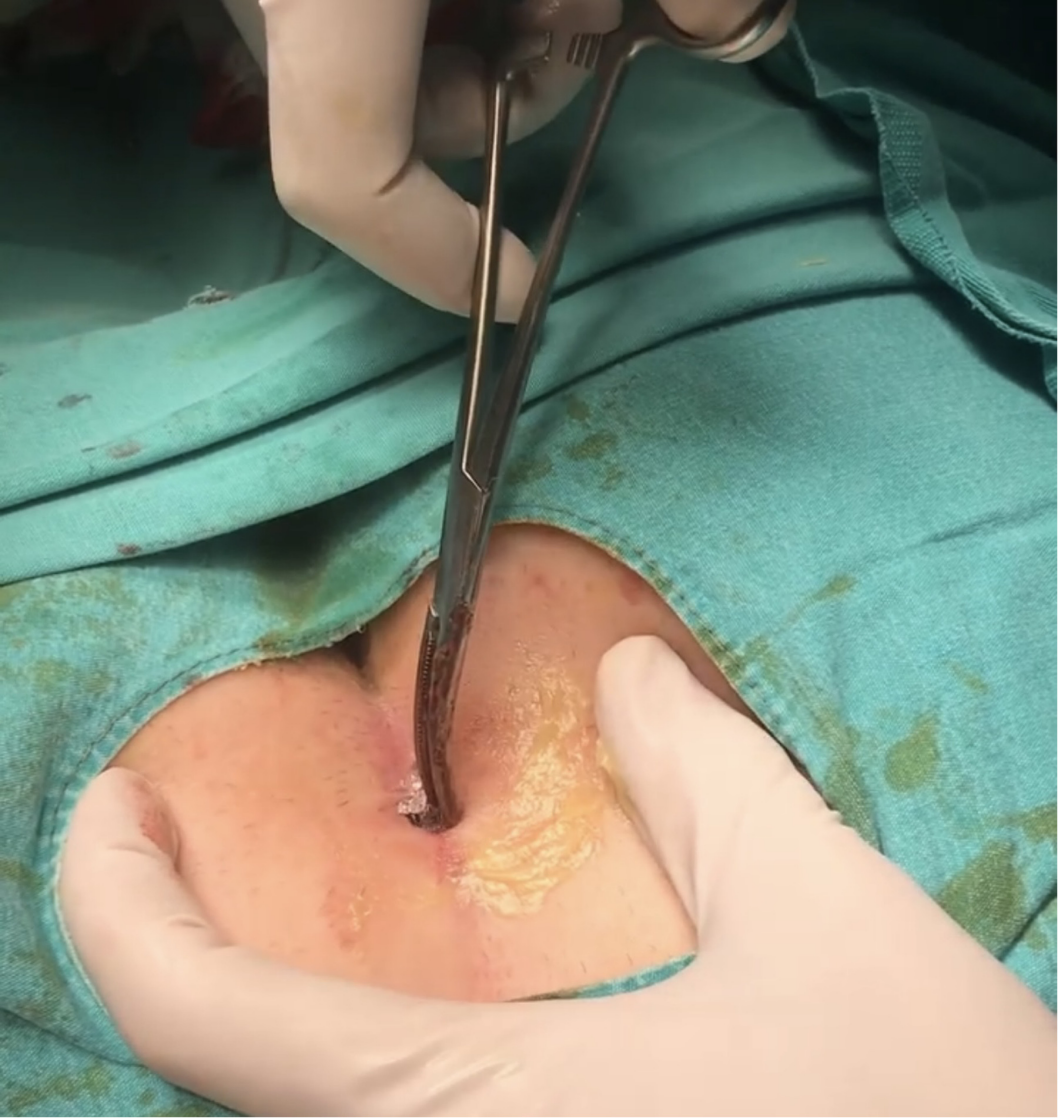
Insertion of crystallized phenol into the enlarged sinus opening.

Crystallized phenol was introduced into the sinus cavity with the help of a surgical clamp and left in place for approximately 3–5 min to ensure adequate destruction of the sinus walls. The procedure was repeated as necessary until the sinus walls were sufficiently cleaned. Finally, the sinus cavity was packed with sterile moist gauze (Figures 1,2).

### 2.4 Postoperative care and follow-up

**Figure 3:**
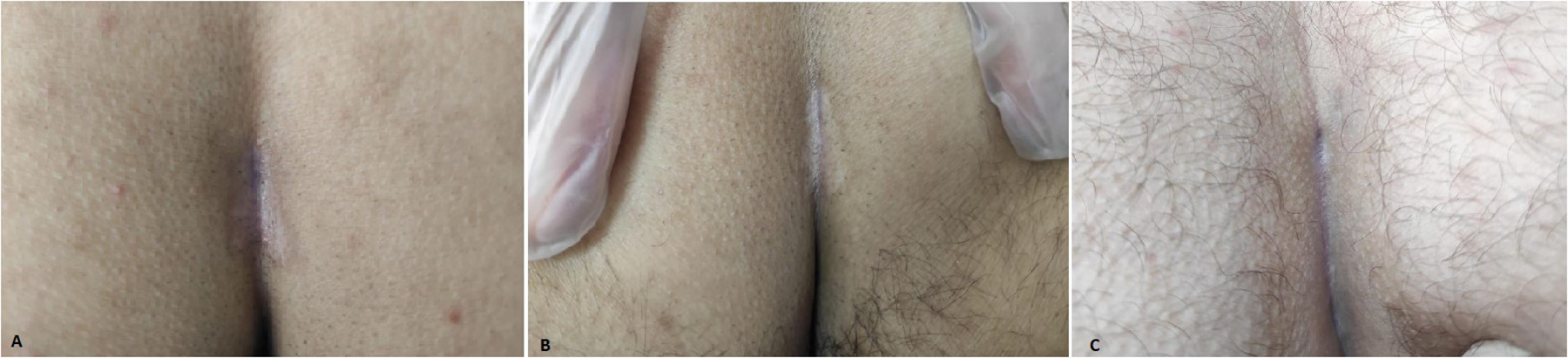
Postoperative appearances of three different patients at 6 months following the procedure, demonstrating closure of the sinus tracts with scar tissue.

Patients were discharged on the same day of the procedure with instructions for wound care and were prescribed analgesics. Antibiotics were provided only for infected cases. Activity restrictions, such as driving or sitting, were not imposed. Follow-up evaluations were scheduled at postoperative days 1 and 7, and monthly thereafter for up to 6 months. Lack of symptoms, including discharge, pain, and erythema, was considered indicative of clinical success. Recurrence was defined as the reappearance of symptoms after an asymptomatic period. Additionally, patients were advised to continue hair removal methods, such as depilatory creams or laser hair removal, postoperatively to reduce the risk of recurrence (Figure 3).

### 2.5 Statistical analysis

All data were analyzed using SPSS (Statistical Package for the Social Sciences) software for Windows (v21.0; IBM, Armonk, NY, USA). Individual and aggregate data were summarized using descriptive statistics, including mean, standard deviations, medians (min-max), frequency distributions, and percentages. The normality of data distribution was verified by the Kolmogorov–Smirnov test. Variables with a normal distribution were compared using the Student's *t*-test. For variables that were not normally distributed, the Mann–Whitney *U* test and Kruskal–Wallis test were used for group comparisons. Categorical variables were analyzed using the Chi-square test. *P*-values < 0.05 were considered statistically significant.

## 3 Results

The 28 patients included in this study consisted of 17 females (60.7%) and 11 males (39.3%). The mean age of the sample group was 15.04 ± 1.40 years (range: 11–17 years) (Table 1).

**Table 1:** Descriptive characteristics.

Parameter	Value	Confidence intervals
Total patients	28	
Gender (F/M %)	17 (60.7%), 11 (39.3%)	
Mean Age	15.04 ± 1.40 years (Range: 11–17)	CI: (14.52, 15.56)
Mean Height	165.46 ± 8.15 cm	CI: (162.44, 168.48)
Mean Weight	68.11 ± 17.11 kg	CI: (61.77, 74.45)
Mean BMI	24.72 ± 5.24 kg/m² (Range: 18.6–43.0)	CI: (22.78, 26.66)
BMI > 25 kg/m²	9 patients (32.1%)	
BMI < 25 kg/m²	19 patients (67.9%)	

BMI, body mass ındex; F, female; M, male; CI, confidence ıntervals.

The average number of orifices was 1.75 ± 0.8 (range: 1–4). Thirteen patients (46.4%) had a single sinus opening, while 15 patients (53.6%) had multiple sinus openings. The orifice location was at the midline in 82.1% (*n* = 23) of the cases, while 10.7% (*n* = 3) were right lateral, and 7.1% (*n* = 2) were left lateral. According to the evaluation of anthropometric measurements obtained from the cases, the mean height was 165.46 ± 8.15 cm, the mean weight was 68.11 ± 17.11 kg, and the mean BMI was 24.72 ± 5.24 kg/m² (range: 18.6–43.0). It was determined that 32.1% (*n* = 9) of the sample group had a BMI above 25 kg/m², while 19 (67.9%) had a BMI below 25 kg/m². Pre-procedural abscess was observed in 78.6% (*n* = 22) of the cases. The mean number of courses per patient was 3.32 ± 1.49 (range: 1–7) (Table 2).

**Table 2:** Clinical findings.

Clinical finding	Details	Confidence intervals
Average number of orifices	1.75 ± 0.8 (range: 1–4)	CI: (1.46, 2.04)
Single sinus opening	13 patients (46.4%)	
Multiple sinus openings	15 patients (53.6%)	
Orifice location	Midline: 23 (82.1%), Right lateral: 3 (10.7%), Left lateral: 2 (7.1%)	
Pre-procedural abscess	22 patients (78.6%)	
Mean number of session	3.32 ± 1.49 (range: 1–7)	CI: (2.77, 3.87)
Phenol Procedure session	1 session: 1 patient 2 session: 6 patients 3 session: 15 patients 5 session: 3 patients 6 session: 1 patient 7 session: 2 patients	

CI, confidence ıntervals.

Phenol treatment was administered as follows: one course for 1 patient, two courses for 6 patients, three courses for 15 patients, five courses for 3 patients, six courses for 1 patient, and seven courses for 2 patients. In our study, the mean follow-up period was 85.5 ± 6.20 days, and the average healing time was determined to be 8.5 ± 6.2 days. Additionally, phenol-related complications (skin burns, cellulitis, painless contact dermatitis) were observed in four patients (14.3%). In the initial evaluation following phenol procedure, recurrence was noted in 21.4% (*n* = 6) of the cases. In one of these cases, recurrence was observed after a single treatment course. Additionally, during follow-up, persistent discharge was observed in 2 patients who received two treatment courses, 3 patients who received three treatment courses, and 1 patient who received seven treatment courses. This was considered as unsuccessful phenol therapy, and surgical excision was planned for these patients. In addition, pre-surgical phenol therapy was administered to 5 patients (17.9%), while post-surgical phenol therapy was administered to 4 patients (14.3%) (Table 3).

**Table 3:** Treatment outcomes.

Treatment outcome	Result	Confidence intervals
Mean follow-up period (Day)	85.5 ± 6.20	CI: (83.21, 87.79)
Average healing time (Day)	8.5 ± 6.2	CI: (6.21, 10.79)
Phenol-related complications	4 (14.3%)	
Recurrence rate	6 (21.4%)	
Unsuccessful phenol procedure	6	
Pre-surgical phenol procedure	5 (17.9%)	
Post-surgical phenol procedure	4 (14.3%)	
Complete closure & success rate	21 (75.0%)	
Total treatment success (Pre/Post-Surgical Phenol Procedure)	23 (82.1%)	
Recurrence after surgery	3 (10.7%)	
No ımprovement & persistent discharge	2 (7.1%)	

CI, confidence ıntervals.

No statistically significant relationship was found between recurrence and BMI (>25 kg/m²), number of orifices (more than one), or the number of treatment courses in the cases (*p*-values = 0.397, 0.418, and 0.772, respectively). At the end of the total follow-up period, it was observed that phenol treatment courses resulted in complete closure of the sinus opening and successful outcomes in 75.0% (*n* = 21) of the patients. Additionally, total treatment success was observed in 82.1% (*n* = 23) of the cases following pre- and post-surgical phenol procedure. Recurrence was observed in 10.7% (*n* = 3) of the cases following surgery, while 7.1% (*n* = 2) showed no improvement at any stage, and surgical treatment was planned due to persistent discharge. In these patients, inadequate hygiene, insufficient debridement, and hair growth were considered the potential reasons for the lack of healing (Table 4).

**Table 4:** Recurrence rate.

Association factor	*P*-value*
Recurrence & BMI relation	0.397
Recurrence & number of orifices	0.418
Recurrence & number of treatment courses	0.772

* Chi-square test. *P*-values < 0.05 were considered statistically significant.

## 4 Discussion

PSD is traditionally recognized as a condition predominantly affecting young males, with studies reporting a male-to-female ratio of approximately 4:1 (6). The peak incidence of PSD generally occurs during the late adolescent and early adult years, correlating with increased hormonal activity, hair growth, and sweat gland function. However, recent studies suggest a shifting epidemiology, with an increasing prevalence among females and younger age groups. Supportively, Torun and Subaşı reported that females constituted 29% of PSD cases (7). Studies focusing on adolescent populations further reveal that the disease frequently manifests during the post-pubertal years. Ankersen et al. documented a median age of 17.2 years in adolescents with PSD, with males still constituting the majority of cases (84%) in their cohort (8). Similarly, a systematic review by Luedi et al. confirmed significant regional variations in the male-to-female ratio, with females accounting for up to 39% of cases in certain regions, particularly where lifestyle factors such as obesity and sedentary behavior are prevalent. In our study, we observed a predominance of female patients (60.7%) and a mean age of 15.04 years, which diverges from the traditionally male-dominant epidemiology. This discrepancy may reflect regional variations, shifts in risk factor profiles, or improved detection and reporting among female patients. The younger mean age aligns with existing literature emphasizing the disease's prevalence in adolescence but highlights the need for further research into gender-specific risk factors.

BMI is a recognized risk factor in PSD, with numerous studies linking higher BMI to increased disease prevalence and postoperative complications. Akbulut et al. reported that higher BMI levels were significantly associated with early postoperative complications in PSD patients (*p* < 0.001), although no direct relationship with recurrence rates was established (9). Similarly, Halleran et al. identified obesity (BMI ≥ 95th percentile) as an independent risk factor for PSD recurrence in their pediatric cohort (10). A study by Torun and Subaşı also highlighted obesity as a contributing factor to disease recurrence (7). Additionally, Hajiesmaeili et al. reported that 52% of obese patients with PSD experienced postoperative wound infections (11). In our study, the mean BMI was 24.72 ± 5.24 kg/m², with 32.1% of patients classified as overweight or obese (BMI > 25 kg/m²). These findings consisted closely with the results of Akbulut et al. and Torun & Subaşı, further supporting the association between higher BMI and PSD risk. In addition, recurrence rates were higher in patients with elevated BMI (44.4% in BMI > 25 vs. 10.5% in BMI < 25), although this difference did not reach statistical significance. These findings suggest a potential trend linking obesity to recurrence.

The number and localization of sinus openings are significant factors that may influence recurrence in PSD. Multiple studies have demonstrated that a greater number of sinus openings correlates with higher recurrence rates. Garg and Yagnik reported that patients with multiple sinus openings had a significantly higher recurrence compared to those with a single sinus opening (12). Similarly, Meinero et al. found that the presence of multiple sinus openings was independently associated with higher recurrence rates, likely due to incomplete excision or inadequate debridement (13). Localization of sinus openings has also been implicated as a factor in recurrence. Midline openings are considered particularly prone to recurrence, as they are subject to repeated mechanical irritation and moisture accumulation. Esposito et al. highlighted that midline external openings were associated with delayed healing and higher recurrence compared to off-midline openings (14). In a cohort study by Gallo et al., the mean number of sinus openings in PSD patients was 2.41, with midline openings accounting for 53.1% of cases. Patients with multiple openings demonstrated longer wound healing times and a higher likelihood of recurrence, particularly when the openings were concentrated along the midline (15). In accordance with these data, the average number of sinus openings was 1.75 ± 0.8, with 46.4% of patients presenting a single sinus opening and 53.6% having multiple openings in our study. The majority of sinus openings (82.1%) were located at the midline. Unlike previous studies, no statistically significant relationship was observed between recurrence and the number of openings (*p* = 0.418). This could be attributed to the standardized phenol treatment protocol employed in our study, which also included thorough debridement and close postoperative follow-up to mitigate recurrence risk.

Phenol treatment has gained attention as a minimally invasive alternative to traditional surgical methods for PSD. Its efficacy in reducing recurrence rates and complications has been well-documented, with success rates ranging from 71% to 93% in various studies (4, 16). Compared to surgical techniques such as excision and primary closure, phenol treatment offers advantages like shorter procedure times, reduced hospitalization, and quicker recovery (17). However, recurrence remains a concern, especially in cases requiring multiple procedures (18). Supportively, in a randomized clinical trial by Şengül et al., phenol treatment showed a recurrence rate of 8%, comparable to the 10% observed with excision and primary closure, but with significantly shorter recovery times and fewer complications. Researchers documented a complication rate of 12%, which included minor skin burns and cellulitis (4). Similarly, in another study by Kargın et al. reported a 71.5% success rate with phenol treatment, emphasizing its effectiveness even in recurrent cases (16). Another study by Sozuer et al. reported a complication rate of 1.4%, primarily consisting of transient dermatitis and minor infections, further supporting the efficacy and safety of phenol as a treatment option (19). Despite these promising outcomes, factors such as BMI, multiple sinus openings, and poor compliance were associated with higher recurrence rates (18). Consistently in present study, the phenol-related complications such as skin burns, cellulitis, and contact dermatitis occurred in 14.3% of patients. Recurrence was observed in 21.4% of cases, aligning with recurrence rates reported in other studies. Interestingly, the success rate was 75% after phenol treatment alone and increased to 82.1% with the inclusion of pre- and post-surgical phenol procedure. The higher recurrence rates in our cohort could be attributed to factors like inadequate debridement, poor postoperative care or poor compliance, as noted in previous studies (16, 17).

The addition of autologous platelet-rich plasma (PRP) injections to crystallized phenol treatment has been shown to significantly improve healing time, cosmetic outcomes, and overall success rates in pilonidal sinus disease. This combined approach has been reported as a safe and effective treatment option for pediatric patients (20).

This study has several limitations. Firstly, the small sample size restricts the generalizability of the results. Additionally, the lack of comparison between patients with similar characteristics and open cases is a significant issue, as it limits the ability to fully evaluate the effectiveness of the treatment in a diverse patient population. The heterogeneity of treatment sessions and methods used further complicates the interpretation of the results, as a more uniform treatment approach would yield more reliable conclusions. Moreover, the retrospective design of the study means there was no randomization in patient selection, which may introduce selection bias. Another limitation is the failure to consider other potential factors influencing treatment outcomes, such as hygiene, wound care, and other individual patient variables. Future studies with larger sample sizes and a prospective design could provide more robust and generalizable findings.

While phenol treatment shows promise as a minimally invasive option for managing PSD, the results in this study are limited by a relatively short follow-up period and the need for multiple treatments in some patients. Therefore, further studies with longer follow-up periods and comparisons to established procedures are necessary to fully assess its long-term efficacy and success rates.

## 5 Conclusions

The success rates of phenol treatment are lower compared to primary surgical and flap techniques. However, its non-invasive nature and the risk of recurrence associated with surgical treatments suggest that phenol can be safely used as a first-line treatment for adolescents. In this study, although the sample size is small, we aimed to demonstrate the efficacy of phenol, as it offers a less invasive option for pediatric patients. To assess its long-term effectiveness and success rates, further studies with longer follow-up periods and comparisons with established procedures are needed.

## Data Availability

The original contributions presented in the study are included in the article/Supplementary Material, further inquiries can be directed to the corresponding author/s.
